# Supplementary material for: The genome sequence of ash, Fraxinus excelsior L. (Lamiales: Oleaceae)
Source: Wellcome Open Res. 2026 Oct 5;11:803. [Version 1] doi: 10.12688/wellcomeopenres.28044.1 (PMC13743841; doi:10.12688/wellcomeopenres.28044.1)
Supplement: Peer review history

# Reviewer report: Reviewer response for version 1

**DOI:** 10.21956/wellcomeopenres.30850.r173182

**Published:** 2026-11-06

**Authors:** Mir Muhammad Nizamani

**Affiliations:** 1 Shantou University, Shantou, China

The manuscript presents a high-quality, haplotype-resolved genome assembly of *Fraxinus excelsior*, an ecologically and economically important tree species threatened by ash dieback and the emerald ash borer. The rationale for genome sequencing is clearly explained, and the authors employ appropriate sequencing, assembly, and quality-assessment methods. The reported assembly metrics demonstrate strong completeness and contiguity, making this a valuable genomic resource.

 However, a few points require clarification:

1. The difference between the flow-cytometry genome-size estimate (920 Mb) and the k-mer-based estimate (767.70 Mb) should be briefly discussed.
2. Table 1 indicates that post-shearing quality-control information was not recorded for one PacBio library. The authors should clarify this omission.
3. The genome annotation is described as forthcoming. Its current availability should be clarified, with an accession or repository link provided if available.
4. A brief quantitative comparison with previously published *F. excelsior* genome assemblies would better demonstrate the improvements achieved.

Overall, the manuscript is scientifically sound, and the reported genome assembly represents a useful contribution to plant genomics and conservation research. The identified concerns are primarily related to reporting clarity and reproducibility rather than fundamental methodological weaknesses. **I recommend minor revision**, with the above points addressed before indexing.

Are the datasets clearly presented in a usable and accessible format, and the assembly and annotation available in an appropriate subject-specific repository?

Partly

Are sufficient details of the sequencing and extraction, software used, and materials provided to allow replication by others?

Partly

Are the rationale for sequencing the genome and the species significance clearly described?

Yes

Are the protocols appropriate and is the work technically sound?

Yes

Reviewer Expertise:

Plant genetics and breeding, plant genomics, biodiversity and conservation, plant pathology, plant–microbe interactions, molecular biology, bioinformatics, and ecological modelling.

I confirm that I have read this submission and believe that I have an appropriate level of expertise to confirm that it is of an acceptable scientific standard.

# Reviewer report: Reviewer response for version 1

**Published:** 2026-10-05

**Authors:** Automated Benchmarking

**Affiliations:** 1 Wellcome Sanger Institute, Hinxton, UK

As this article is a Genome Note, Automated Benchmarking has been used to determine whether the reported assembly passes four defined checks established by the Earth BioGenome Project: the raw and assembled data are publicly available, the Earth BioGenome Project three-part metric is met, the k-mer completeness exceeds 90%, and the BUSCO score passes the stated threshold. For Fraxinus excelsior (daFraExce3), all four checks passed. Data available: pass. ENA study PRJEB81013, raw-read accessions ERR13800547, ERR13800546, ERR13802685, and ERR13802686, and assembly accessions GCA_965226085.2 and GCA_965226315.2 are public. Earth BioGenome Project three-part metric: pass. Haplotype 1 is reported as 6.C.Q63, meeting the 6.C.Q40 reference standard. k-mer completeness: pass. The combined haplotype assemblies have 99.44% k-mer completeness, exceeding the Earth BioGenome Project target of 90%. BUSCO: pass. Haplotype 1, which is proposed as the reference assembly for this species, has 99.2% complete BUSCOs (76.7% single-copy and 22.5% duplicated). BUSCO software version: 6.0.0; lineage dataset: eudicots_odb10. It meets the plant-specific target of greater than 90% complete BUSCOs. This decision uses values reported in the Genome Note and public accession records.

Reviewer Expertise:

NA

I confirm that I have read this submission and believe that I have an appropriate level of expertise to confirm that it is of an acceptable scientific standard.
